# Challenges and opportunities in the uptake of simulation in healthcare education in the developing world: a scoping review

**DOI:** 10.12688/mep.20271.1

**Published:** 2024-05-24

**Authors:** Faisal W. Ismail, Khairulnissa Ajani, Syed Mujtaba Baqir, Ahmed Nadeem, Rayyan Qureshi, Pammla Petrucka

**Affiliations:** 1 Centre for innovation and Medical Education, The Aga Khan University, Karachi, Sindh, Pakistan; 2 Department of Nursing, The Aga Khan University, Karachi, Sindh, Pakistan; 3 Department of Medicine, The Aga Khan University, Karachi, Sindh, Pakistan; 4 Medical College, The Aga Khan University, Karachi, Sindh, Pakistan; 5 Department of Nursing, University of Saskatchewan, Saskatoon, Saskatchewan, Canada

**Keywords:** education, healthcare, simulation, opportunities, challenges

## Abstract

**Background:**

Simulation is increasingly being adopted by healthcare educators throughout the developed world as it offers a safe environment to practice skills. While there is literature on learning via simulation in healthcare in the developed world, more studies are required to investigate the factors influencing this approach in the developing world.

**Objective:**

This scoping review highlights the key factors that act as deterrents as well as encouragement to the uptake of simulation as a teaching methodology in healthcare education in developing countries.

**Design:**

The MEDLINE (via OVID, using keywords and MeSH in OVID), and PubMed (via NCBI using MeSH), and CINAHL databases were searched between January 2000 and January 2024 for research articles published in peer reviewed English language journals using a combination of keywords.

**Results:**

A total of 48 articles were included in the final analysis. Challenges and opportunities were divided into professional, academic, and resource-based factors, and their individual sub-themes. The main challenges reported were the lack of a contextual curriculum, content heavy curricula, dearth of trained simulationists and cost of simulators. Performance anxiety was an important challenge reported by both trainers and trainees. Main opportunities were an interest in adopting simulation-based education from both trainers and trainees, and the opportunity to improve patient safety and quality of education. Other findings were that academic leadership and faculty show interest and urgency to adopt simulation in curricula and allocate funds for this. Facilitators need to be provided with protected time to become simulationists. Local manufacturers need to be sourced for simulators, and transfer of technology and expertise needs to be negotiated.

**Conclusion:**

Simulation needs to be looked at from the lens of not only education, but more importantly of patient safety in developing countries to allow simulation-based education to be mainstreamed in health professions education in low- and middle-income contexts.

## Introduction

Simulation is increasingly being adopted by healthcare educators throughout most of the developed world as it offers a safe and controlled environment in which to train a particular set of clinical skills. The field of medicine requires pre-service practitioners to be adept with complex and often invasive procedural skills, while learning them without causing harm to patients ^1^. Simulation-enabled health sciences education aims to reproduce clinical scenarios using simulators. This approach provides a vital link between theoretical medicine and the practical application of management guidelines, preparing pre-service practitioners for real-life high-pressure situations. Simulation is therefore immensely beneficial to students and faculty as it decreases the burden on human resources, prevents wastage of clinical equipment, provides a safe environment, and enables effective learning without compromising patient safety ^2^.

The application of simulation to healthcare education is widespread in the developed world. For example, in Hungary, the national simulation network includes three universities and 16 hospitals and has a formalized national protocol for simulation-related research, 3D-printing technology, and virtual/augmented reality utilization in health sciences education ^3^. While there is literature on experiences from learning via simulation in healthcare in the developed world, more studies are required to investigate the factors influencing the advancement and embedding of this approach in the developing world ^4^. As simulation in healthcare education requires immense pedagogical, financial, and logistical resources, a detailed analysis of the barriers and facilitators is imperative to inform how low to middle-income regions can implement sustainable simulation-enabled medical learning environments.

Scoping reviews are being used increasingly being utilized to review and understand complex research evidence in areas where there is a dearth of randomized controlled trials ^5^. It enables researchers to include a range of different study designs that address questions beyond those related to the effectiveness of a particular intervention and provide a structured approach to the collection and organization of the existing evidence base related to a precise research question ^6^. The PRISMA ScR guidelines were used as the guiding document in the conduct of this study ^7, 8^.

## Methods

For the purpose of our scoping review on the challenges and opportunities to the uptake of simulation in healthcare education in low- and middle-income contexts, we searched for research articles published between January 2000 and January 2024 using PubMed (via NCBI using keywords), EBSCO CINAHL Plus (using keywords), EBSCO Dentistry and Oral Sciences Source (via keywords), Wiley Cochrane Library (via keywords) and ProQuest Theses and Dissertations Database (via keywords). Only articles in peer-reviewed journals in English were considered. Table 1 shows the combination of keywords used and summarizes the selection process for the articles.

**Table 1.** The combination of keywords used.

Barriers OR Impedance OR Hurdles OR Challenges OR Hesitance OR Inhibition OR Difficulty OR Reluctance OR Hindrance OR Limitation OR Resistance OR Refusal Facilitators OR Factors OR Influencers OR Influences OR Promoters OR Causes OR Reasons OR Elements Uptake OR Acceptance OR Compliance OR Approval OR Agreement OR Embracing OR Willingness OR application OR Endorsement OR Amenableness Simulation OR Recreation OR Technology OR Simulators OR Medical Simulation OR Simulation- based Medical Education OR Simulation-based Healthcare education OR Simulation-based Heath Professions Education OR Simulation-based Training OR Simulation-based Interprofessional Training OR Simulation-based Inter-professional Training OR Medical Education OR Simulation- based Interprofessional Healthcare education OR Simulation-based Inter-professional Healthcare Education Health Professions Education OR Health Professions Training OR Healthcare Education OR Healthcare Training OR Medical Education OR Medical Training OR Medical Skills OR Health Professional Skills OR Health Professional Training OR Interprofessional Medical Education OR Inter-professional Medical Education OR Inter-professional Medical Training OR Interprofessional Healthcare Education PR Inter-professional Healthcare Education OR Inter- professional Healthcare training OR Healthcare Professional Skills OR Healthcare Training OR Healthcare Skills Teaching Strategy OR Strategy OR Methodology OR Teaching Method OR Teaching Methodology OR Training Strategy OR Training Method OR Training Methodology OR Educating Strategy OR Educating Method OR Educating Methodology OR Training Approach OR Teaching Approach OR Approach Countries with Developing Economy OR Low-middle-income countries OR Low-Middle Income Countries OR Low Middle-Income Countries OR Low Middle Income Countries OR Lower-Middle- Income Countries OR Lower Middle-Income Countries OR Lower-Middle Income Countries OR Middle Income Countries OR Middle-Income countries OR Medium Income countries OR Medium-Income Countries OR Developing Countries OR Developing World OR Developing Nations OR Underdeveloped Countries OR Underdeveloped World OR Underdeveloped nations OR Economically Developing Countries OR Economically Developing Nations

This search produced 906 articles in total which were organized in ENDNOTE™ (Version 21, Zotero is a tool that can be used as a freely available alterantive to ENDNOTE) based on reference links, sources, and abstracts. The inclusion and exclusion criteria, as shown in Table 2, were extensively discussed amongst the team.

**Table 2.** The inclusion and exclusion criteria used.

Reviewed by 2 independent dyads of researchers using the following:
**Inclusion Criteria:** 1. Studies highlighting barriers &/or facilitators to uptake of healthcare simulation. 2. Studies conducted in developing nations. 3. Studies related to the delivery of simulation-based health sciences education
**Exclusion Criteria:** 1. Studies/reviews highlighting benefits of simulation. 2. Studies describing the process of the simulation

Of the 906 articles retrieved after the first search, 556 articles were excluded after the first iteration as they did not meet the inclusion or exclusion criteria; included reasons were that papers were not from the context of a developing context, advocated the use of simulation as a teaching strategy, not identifying barriers or facilitators, or discussed the role of simulation in non-health care settings.

The four researchers split into independent dyads and divided the remaining 350 articles amongst themselves to select ones which distinctly focused on the research question. The fifth researcher was approached for a decision in case of a disagreement.

**Figure 1.**
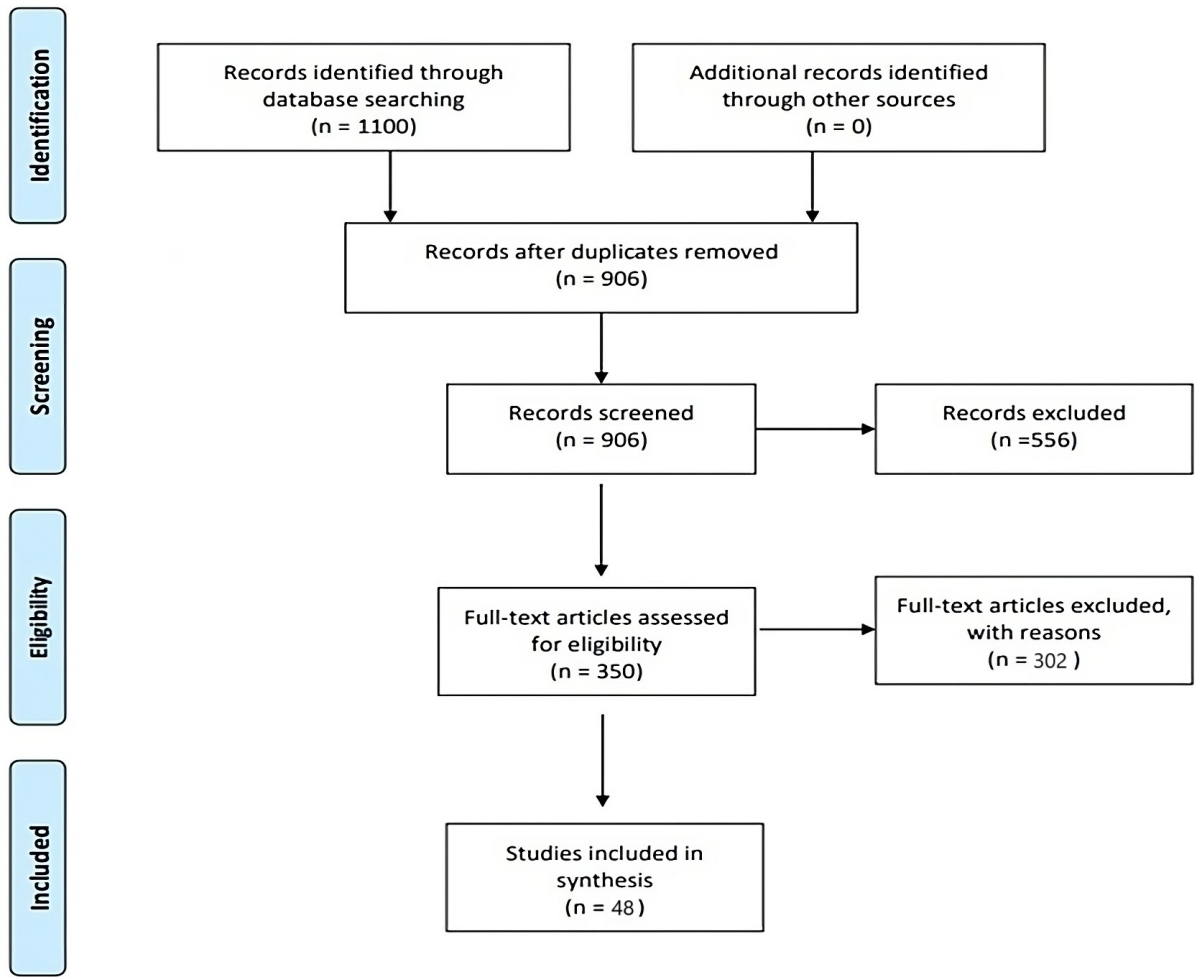
Study selection process.

A total of 302 articles were excluded after this iteration as they did not include the barriers and facilitators, often dealing with delivery of specific simulations or use of simulation in healthcare settings, but not medical education. Thus, 48 articles were selected for final analysis which contributed to identification of themes emerging and organization into categories to address the aim of our scoping review. The organization is illustrated in Figure 1.

Using Microsoft Excel™ (2021 Version), a spreadsheet was created to organize the data from the 48 articles with the following headings: Author and Year of Publication, Title, Keywords, Objective, Methodology, Study Population, Key Findings of Barriers, and Facilitators that increase the uptake of simulation as a strategy ( Table 3).

**Table 3.** Results of the individual articles that were selected to be used in this Scoping Review.

S#	Author & Year	Title	Methodology	Study objective(s)	Study population	Facilitator in the uptake of simulation	Barrier in the uptake of simulation
1	Martinerie L. (2018)	Health care simulation in developing countries and low-resource situations	Literature review	To analyze health care simulation experiences in developing countries.	Interdisciplinary, under and postgraduate	1.Sustainability of programs. 2. Student interest.	1. Lack of financial and human resources, ongoing armed conflicts, and poor health infrastructure logistics and time constraints. 2. Lack of context specific curriculum.
2	Salman H. (2021)	Most significant barriers and proposed solutions for medical schools to facilitate simulation-based undergraduate curriculum in OBGYN	Review	To discuss the relevance and necessity of a simulation-based undergraduate curriculum in obstetrics and gynecology. What are the biggest obstacles that medical schools face in making the most of simulation-based learning, and how can they be overcome?	UG medical students	1.Use of low-cost simulators 2.Use of SP`s 3.Faculty development.	1. Cost. 2. Lack of trained staff. 3. Lack of context specific curriculum.
3	Andreatta P. (2017)	Healthcare simulation in resource-limited regions and global health applications	Editorial review			1.Carefully designed culturally sensitive simulation-supported encounters and mastery learning-centered instruction.	1. Cost. 2. Infrastructure 3. Contextual curriculum. 4. High number of students.
4	A. Wang. (2020)	Establishing a Low-Resource Simulation Emergency Medicine Curriculum in Nepal	Interventional study	To study the effects of introducing a simulation curriculum in a low-resource environment.	Medical officers and students in the ED		1. Cost. 2. Lack of contextually relevant curriculum. 3. Content heavy curriculum.
5	A. R. L. Rule. (2017)	The Call and the Challenge of Pediatric Resuscitation and Simulation Research in Low-Resource Settings	Randomized interventional study	To evaluate skill retention after an initial Helping babies, breathe simulation training	Healthcare staff		1. Lack of protected education time and resources. 2. Lack of familiarity with simulation. 3. Cultural differences in giving feedback. 4. Poorly trained facilitators. 5. High acuity and clinical volume.
6	A. Kesrouani. (2019)	Integrating obstetrical simulation into the medical curriculum: one more gap in women's health for low-income countries	Literature Review	Low-income countries do not have well-established simulation centers; Simulation-based learning yields many benefits comparable to found in higher income countries.	-	1.Understanding that simulation based curricula yield the same benefits in developing countries as in developed ones. 2. Increased use will lead to more uptake of simulation in these regions.	1. Rigid curriculum, lack of flexibility. 2. Low-income countries do not have established surgical simulations, centralization, and cooperation amongst the educational institutions and local and regional hospitals for maintenance of medical educational practices and financial supplementation. 3. Lack of competent trainers.
7	K. E. Flaherty. (2020)	The state of emergency medical technician education in Ghana	Observational study	To understand the views of students and faculty on the current emergency medical technician curriculum and assess barriers to integration of simulation-based learning	Faculty and EMT students	1. Overwhelming favourable opinion towards simulation of students. 2. High engagement from students and faculty.	1. Language barriers. 2. Resource concerns. 3. Lack of trained staff. 4. Limited leadership support and inclination to modernise curricula.
8	P. A. Afulani. (2020)	Provider knowledge and perceptions following an integrated simulation training on emergency obstetric and neonatal care and respectful maternity care: A mixed-methods study in Ghana	Prospective cross-sectional study with a mixed method research design	To examine the effectiveness of integrated simulation training on emergency obstetric and neonatal care and respectful maternity care on providers' knowledge and self-efficacy, and to assess providers' perceptions of the integrated training.	Forty-three Maternity providers	1. Providers recognized added valued in the training. 2. Prior training on use of simulator.	1. Small duration of simulation-based training intervention. 2. Not enough sessions per year to allow expertise to develop.
9	M. D. Traynor. (2021)	Surgical Simulation in East, Central, and Southern Africa: A Multinational Survey	Descriptive, survey	To characterize the current state of surgical skills simulation in East, Central, and Southern Africa and determine residents’ perception and future interest in such activities.	76 surgical trainees	1.Eagerness of participants to accept simulation as a tool for education.	1. Lack of suitable tools and models. 2. High costs and funding Issues. 3. Maintenance of facilities. 4. Lack of prior regular use of simulation.
10	Yin Mar OO. (2020)	The application of simulation-based medical education in low- and middle-income countries; the Myanmar experience	Descriptive study	To describe the various modalities of SBME that may be utilized in a LMIC in South East Asia		1.Faculty buy-in 2.Scholarships for faculty development. 3.Partnerships with established sim centres.	1.Resource limitations 2. Personnel availability 3.Language barriers 4.Small existing skill base.
11	R. Shrestha. (2019)	Interdisciplinary in situ simulation-based medical education in the emergency department of a teaching hospital in Nepal	Prospective cross-sectional study with a mixed method research design	To introduce cost-effective in situ simulation (ISS) in the emergency department (ED), to explore its impact on perception and learning experience among multidisciplinary health care professionals and to identify and remediate latent safety threats (LST)	56 healthcare staff in the ED	1. Ability of simulation to increase confidence and provide better understanding.	1. Financial burden. 2. Baseline knowledge about the use simulators low. 3. Performance anxiety of healthcare providers.
12	A. M. Chima. (2018)	Medical Simulation as a Vital Adjunct to Identifying Clinical Life-Threatening Gaps in Austere Environments	Quasi-experimental study	To use simulation to identify areas needing improvement and to rehearse best practice approaches,	Nurse anesthetists	1.Feasibility and value of in situ simulation-based performance assessment.	1. Sustainability 2. Skills transfer in low resource environment. 3. Dearth of skilled trainers due to lack of exposure.
13	P. S. Loh. (2021)	A Developing Nation's Experience in Using Simulation-Based Training as a Preparation Tool for the Coronavirus Disease 2019 Outbreak	Interventional study, followed by debriefing and evaluation.	To describe the preparation in an anesthetic department using simulation-based training over 2 weeks	Healthcare staff in the Anesthesia department	1.Faculty feedback to institution regarding utility of simulation as a teaching method.	
14	R. M. Piryani. (2019)	Simulation-based education workshop: perceptions of participants	Semi-structured quasi-experimental study.	To evaluate perceptions of participants on SBE and an SBE workshop.		1.Overwhelmingly favorable opinion about simulation-based education.	
15	T. M. Renouf. (2018)	Collaborative Development of a Simulation-augmented Health Education Program in Resource-challenged Regions	Literature review	Review of the literature and the authors' experience in developing, delivering, and evaluating sustainable HPE programs in resource-poor regions using simulation		1. Novelty of simulation. 2. Provides opportunity to practice. 3. Promotes patient safety.	
16	E. G. Bing. (2021)	User Experience With Low-Cost Virtual Reality Cancer Surgery Simulation in an African Setting	Interventional, semi-structured interviews	To explore gynecologic oncology trainee learning and user experience using a low-cost VR simulator to learn to perform an open radical abdominal hysterectomy in Lusaka, Zambia.	11 surgical trainees	1.Trainee and trainer enthusiasm for the uptake of simulation. 2.Low cost, indigenously built simulators.	1. Cost. 2. Inadequately trained facilitators.
17	F. Nicolosi. (2018)	Neurosurgical digital teaching in low-middle income countries: beyond the frontiers of traditional education	Literature Review	To introduce digital platforms in resource poor settings as an alternative solution for bridging the gap between Western and poor countries in neurosurgical knowledge.		1. Positive impact on the ability to diagnose, perform procedures, and integrate basic sciences and clinical medicine.	
18	R. Annoh. (2021)	Experiences and Perceptions of Ophthalmic Simulation-Based Surgical Education in Sub-Saharan Africa	A multi-center, multi-country qualitative study	To explore the experiences of trainee ophthalmologists and ophthalmic surgeon educators' use of simulation, and the perceived challenges in surgical training	Twenty-seven trainee ophthalmologists and 12 ophthalmic surgeon educators from six training centers in sub-Saharan Africa	1. Ability to simulate different diseases in the same setting. 2. Opportunity to practice.	1. Cost. 2. Trained facilitators.
19	S. R. Sabzwari. (2017)	Mimicking rashes: Use of moulage technique in undergraduate assessment at the Aga Khan University, Karachi	Observational study	To assess the validity and feasibility of moulage techniques for medical students summative Objective Structured Clinical Examination (OSCE)	Undergraduate medical students	Opportunity to practice skills in different environments.	
20	B. A. Akber. (2021)	Simulated learning in rural community environment: pushing the boundary	Review article	To discuss the concept of creating a novel simulated village set-up within a modern simulation center, to effectively deliver contemporary learning outcomes	-		1. Decreased investment in the healthcare sector. 2. Limited quantitative and qualitative capacity of healthcare staff.
21	S. Taché. (2009)	Addressing gaps in surgical skills training by means of low-cost simulation at Muhimbili University in Tanzania	Interventional study, followed by feedback evaluation	To enhance technical skills in general surgery and emergency procedures for senior medical students by designing and implementing a surgical skills practicum using locally developed simulation models	Thirty-six surgical students		Lack of resources to promote education.
22	N. M. Plana. (2018)	The First Year of Global Cleft Surgery Education Through Digital Simulation: A Proof of Concept	Observational study	To establish a partnership between the academic, nonprofit, and industry sectors for the development of an online virtual surgical simulator for cleft repair.	Surgery residents from 849 countries	1. Free online accessibility of simulator. 2. Technological resources like high-definition intraoperative footage.	Maintenance of simulators.
23	O. Hasan. (2019)	The need for simulation in surgical education in developing countries. The wind of change.	Review article	To study the importance of simulation in surgical education and to question its utility in developing countries.		Interest high to adopt simulation from trainers and trainees.	1. Cost and Internet Issues. 2. Maintenance of infrastructure.3. Standardization of curricula will be required to allow physicians to practice between countries.
24	U. Zubair. (2020)	Surgical resident training in Pakistan and benefits of simulation-based training	Review article	To demonstrate the results of studies comparing the efficacy of trainees trained via the traditional apprenticeship model versus simulator-based training.	-	1. Faculty interest in utilising simulation. 2. Ability to use technology to train.	1. Adequate infrastructure to support SBE. 2. Lack of local industry to manufacture simulators.
25	Nuzhat A. (2014)	Role and challenges of simulation in undergraduate curriculum	Survey	to obtain the opinion of undergraduate medical students and faculty regarding the role of simulation in undergraduate curriculum, the simulation modalities used, and the perceived barriers in implementing simulation sessions	UG medical students Faculty	1.Institutional support 2.Resources 3.Trained staff 4.Adequate duration of sessions.	1. Student motivation to participate. 2. provision of adequate feedback by the staff. 3. Operator expertise.
26	A. Arigbede. (2015)	Use of simulators in operative dental education: experience in southern Nigeria	Cross-sectional study	To determine current practices relating to teaching and learning of dental clinical skills using simulators in southern Nigeria.	final year dental students in southern Nigeria	Less expensive equipment, locally sourced	
27	R. S. Kantar. (2021)	Comprehensive Cleft Care Delivery in Developing Countries: Impact of Geographic and Demographic Factors	Qualitative descriptive study	To analyze the insights of participants and faculty members of Global Smile Foundation's Comprehensive Cleft Care Workshops concerning the barriers and interventions to multidisciplinary cleft care delivery, after stratification based on demographic and geographic factors.	313 participants comprising surgical trainees and faculty	1. Acceptance and enthusiasm of educator and trainee to adopt simulation. 2. Adoption of low-cost simulators in training programs. 3. Hybrid training programs.	
28	S. A. Deganus. (2009)	SYMPTEK homemade foam models for client education and emergency obstetric care skills training in low-resource settings	Descriptive study	To describe the uses, advantages, disadvantages, and development of the SYMPTEK foam models for emergency obstetric care	-	Promotion of local industry to manufacture low-cost simulators.	Cost.
29	F. Mery. (2021)	Reusable Low-Cost 3D Training Model for Aneurysm Clipping	Descriptive, exploratory study	To evaluate a reusable low-cost 3-dimensional printed training model for aneurysm clipping	Thirty-two neurosurgery residents	Manufacturing of low-cost simulators.	
30	A. Okrainec. (2010)	Telesimulation: an effective method for teaching the fundamentals of laparoscopic surgery in resource-restricted countries	Case-control study	To determine the effectiveness of telesimulation for teaching the Fundamentals of Laparoscopic Surgery (FLS) to surgeons in Botswana, Africa.	16 surgical trainees and faculty	Novelty, ease, and opportunity to interact with trainers.	Cost of training and equipment.
31	G. Bediang. (2011)	Relevance and usability of a computerized patient simulator for continuous medical education of isolated care professionals in sub-saharan Africa	Interventional study	To explore the relevance and usability of using a computerized patient simulator as a tool for continuous medical education and decision support for health professionals in district hospitals in Sub-Saharan Africa	88 medical students	Acceptability by students as a good resource.	
32	J. N. Najjuma. (2020)	Stakeholder perceptions about the establishment of medical simulation-based learning at a university in a low resource setting: a qualitative study in Uganda	Qualitative study using focus group discussions (FGDs)	To describe the perceptions of various stakeholders regarding the introduction of SBL methodology into learning at a medical school in Uganda.	Multidisciplinary trainees and faculty	Perception of novelty and innovation by all stakeholders.	1. Old fashioned teaching methodology. 2. Perception of simulation being very expensive.
33	Z. Haroon. (2020)	COVID-19 Era: Challenges and Solutions in Dental Education	Literature review	To review and explore innovative solutions for dental education utilizing manikins and virtual reality/augmented reality (VR/AR)-based simulation devices for skills training.	-	Perception of novelty and innovation.	1. Poor internet connection. 2. Frequent power outages. 3. Scarcity of advanced simulation machines in developing world. 4. Cost.
34	R. Adhikari. (2021)	A mixed-methods feasibility study to assess the acceptability and applicability of immersive virtual reality sepsis game as an adjunct to nursing education	A two-stage sequential mixed-methods feasibility study.	To investigate (1) the impact of IVR sepsis game on pre-registration nurses' self-efficacy and, (2) their perceptions of the acceptability and applicability of IVR sepsis game as an adjunct to nursing simulation education	19 pre-registration nurses	1. Novelty and interesting scenarios to practice. 2. Academic leadership interest in promoting SBE.	
35	M. H. T. Kho. (2018)	Implementing blended learning in emergency airway management training: a randomized controlled trial	Prospective randomized controlled trial	To evaluate the effectiveness of blended learning and simulation in emergency airway management training	30 physicians	1. Excellent acceptability from trainers and trainees. 2. Effective knowledge transfer and self effectiveness.	1. Utilization of low-cost simulators. 2. Increasing investments in the power sector, infrastructure.
36	T. J. McClellan. (2019)	Low-fidelity Paediatric Surgical Simulation: Description of Models in Low-Resource Settings	Descriptive.	To provide a framework to construct simulation models for training opportunities in low-middle-income countries.	-	Easily reproducible in resource-challenged healthcare settings.	1. Cost. 2. Low-cost models suffering from low fidelity.
37	A. Kapoor. (2021)	Simulated Patients for Competency-based Undergraduate Medical Education in Post COVID-19: A New Normal in India	Literature review	To review the need and use of simulated patients; their advantages, limitations and role in students' teaching and assessment.	-	Widely available Resource non-intensive.	Cost.
38	M. C. Morgan. (2018)	Barriers and facilitators to the provision of optimal obstetric and neonatal emergency care and to the implementation of simulation-enhanced mentorship in primary care facilities in Bihar, India: a qualitative study	Interventional study	To explore factors affecting care provision and mentorship, and to improve the quality of care and to maximize the impact of mentoring programs. To explore barriers and facilitators to optimal care provision and to implementation of simulation-enhanced mentorship in PHCs in Bihar	20 Maternity providers in Bihar, India	1. Improved skills and confidence amongst providers. 2. Opportunity for interprofessional training. 3. Increased training frequency 4. Strong mentor-mentee relationship. 5. Administrative support. 6. Nursing supervision and feedback.	1. Human resource shortages. 2. Nurse-nurse hierarchy. 3. Distance between labor and training rooms. 4. Low skill level and resistance to change among mentees. 5. Physical resource shortages. 6. Doctor-nurse hierarchy. 7. Corruption and violence against providers.
39	I. Tjoflåt. (2017)	Implementing simulation in a nursing education programme: a case report from Tanzania	Literature Review	To present a description of, and some reflections around the experience of implementing simulation-based education within a nursing education programme in a low-income context	Undergraduate nursing students	1. Positive student feedback 2. Leadership support and recognition	Time and training required by trainers to become familiar with the equipment.
40	R. M. Nataraja. (2020)	Overview of a novel paediatric surgical simulation-based medical education (SBME) programme in Myanmar	Quasi-experimental study	To use SBME to address some essential paediatric surgery learning needs in a LMIC	Trainees in paediatric surgery	Trainee enthusiasm.	Trainers not adequately trained to deliver SBE.
41	I. Tjoflåt. (2021)	Simulation-based education as a pedagogic method in nurse education programmes in sub-Saharan Africa - Perspectives from nurse teachers	Descriptive, qualitative study	To describe and discuss nurse teachers’ experiences with simulation as a pedagogic method in two educational programmes in low–income countries in sub–Saharan Africa	Nurse teachers in nurse education programmes in Madagascar and Tanzania.	1. Educator buy-in. 2. Appropriate environment.	1. High Number of Students. 2. Challenge of incorporating the method in the setting of busy clinical practice and high patient volumes.
42	R. S. Kantar. (2021)	Perceived Barriers to Comprehensive Cleft Care Delivery: Results From A Capacity-Building Educational Initiative and Implications	Survey	To analyze the perceptions of participants and faculty members in simulation-based comprehensive cleft care workshops regarding comprehensive cleft care delivery in developing countries	313 participants comprising surgical trainees and faculty		Time, effort, and interest required on behalf of the trainers to understand the equipment and operate it.
43	F. Bulamba. (2019)	Feasibility of Simulation-Based Medical Education in a Low-Income Country: Challenges and Solutions From a 3-year Pilot Program in Uganda	Descriptive	To describe the challenges encountered, solutions deployed, and the costs incurred while establishing two simulation centers in Uganda.	-	1. Improvisation of equipment. 2. Customization of low-cost simulation software. 3. Creation of context specific curricula 4. Administrative support. 5. Creation of a simulation fellowship opportunity for local instructors.	1. Costs. 2. Difficulty in procurement of equipment. 3. Lack of context-appropriate curricula. 4. Unreliable power. 5. Limited local teaching capacity. 6. Lack of coordination among user groups.
44	M. Aljahany. (2021)	Simulation-Based Peer-Assisted Learning: Perceptions of Health Science Students	Survey	To evaluate the perceived advantages of simulation-based peer-assisted learning among health professions students and interns and their acceptance of this new concept of learning from a student-instructor.	11 students in various health profession fields	Opportunities to learn from discussions and practice with peers.	
45	Skelton. (2016)	Low-fidelity Simulation to Teach Anesthetists' Non-technical Skills in Rwanda	Randomized Controlled Trial	To examine whether low‐fidelity instructor‐driven simulation can provide effective teaching of anesthetists' non‐technical skills in a developing world context.	Anesthesia Healthcare providers	Opportunity to provide effective teaching using limited resources.	
46	D. Padhya. (2021)	Training of Pediatric Critical Care Providers in Developing Countries in Evidence Based Medicine Utilizing Remote Simulation Sessions	Prospective Observational Study	To determine the feasibility and impact of remote simulation training of international pediatric ICU providers using an electronic decision-making tool.	Pediatric ICU residents and nursing staff.	Flexibility to connect people around the world with audio/video and screen sharing information on a large scale with minimal cost.	1. Language barriers. 2. Limited number of trainers. 3. Limited internet connectivity. 4. Fidelity-the sessions may not completely reflect the actions of these providers in real clinical setting.
47	Puri L. (2017)	Enhancing quality of medical care in low income and middle-income countries through simulation-based initiatives: recommendations of the Simnovate Global Health Domain Group	Scoping review Survey	To consider the role of simulation in LMICs, to directly impact health professions education, measurement, and assessment.		1. Use of low resource simulators 2. Collaborative network 3. Institutional buy in.	
48	Muhumuza, A (2023)	Understanding the barriers and enablers for postgraduate medical trainees becoming simulation educators: a qualitative study.	Exploratory qualitative	To explore the barriers and enablers to engaging PGs as simulation educators for undergraduate medical students and identify key priority areas for consideration prior to implementation of the intervention.	Post graduate trainees, administrative staff and managers at Mbarara University of Science and Technology (MUST) in Uganda.	***1. Favourable departmental attitude*** ***2. Enthusiasm to participate.*** ***3. Awareness of the duties of a simulation educator*** ***4. Departmental sensitization and engagement in simulation activities***	1.Competing time demands. Simulation not integrated into curriculum. 2. Skepticism towards realism in medical simulation ***3. Inadequacy of medical simulation equipment***

## Results

**Figure 2.**
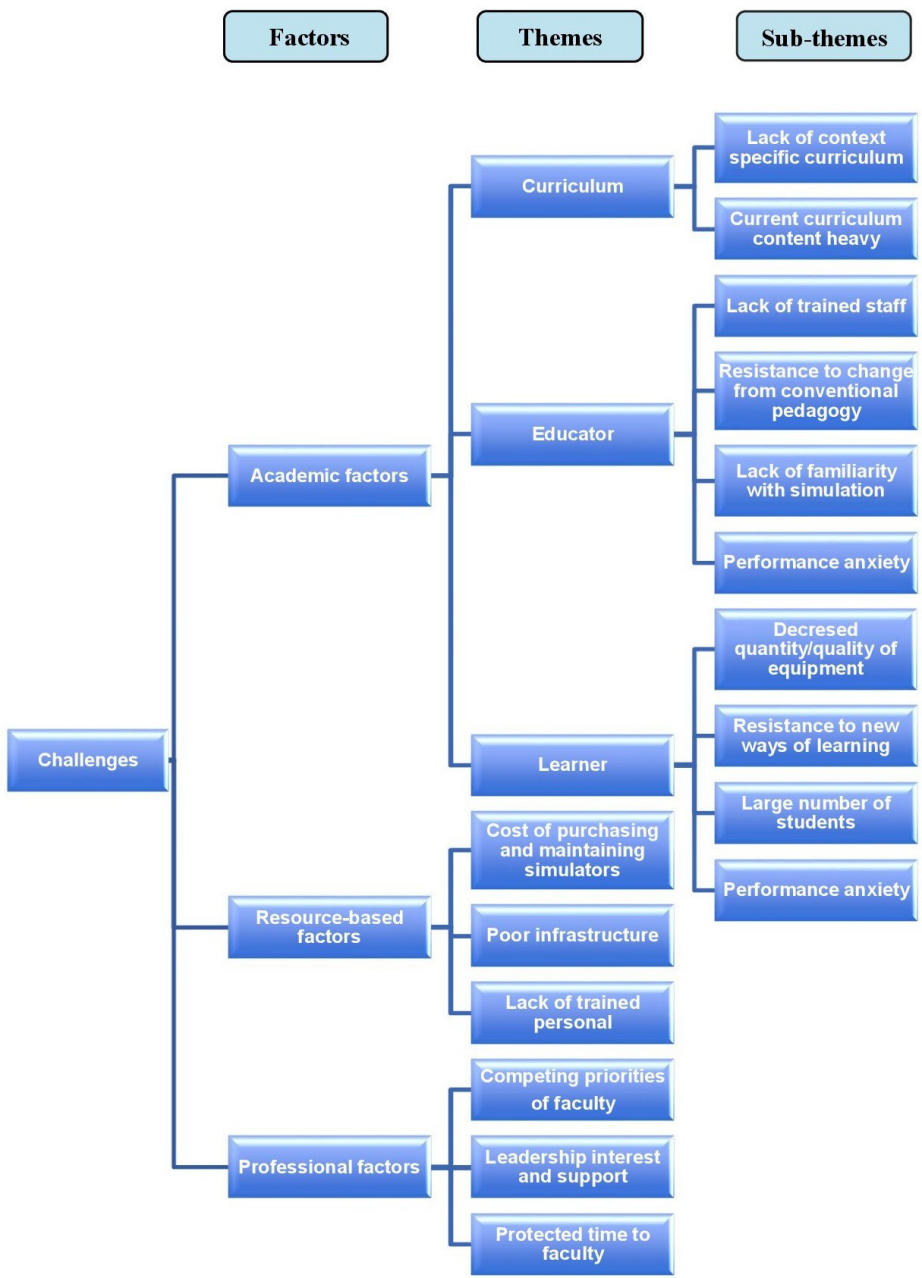
Challenges faced in the uptake of simulation.

**Figure 3.**
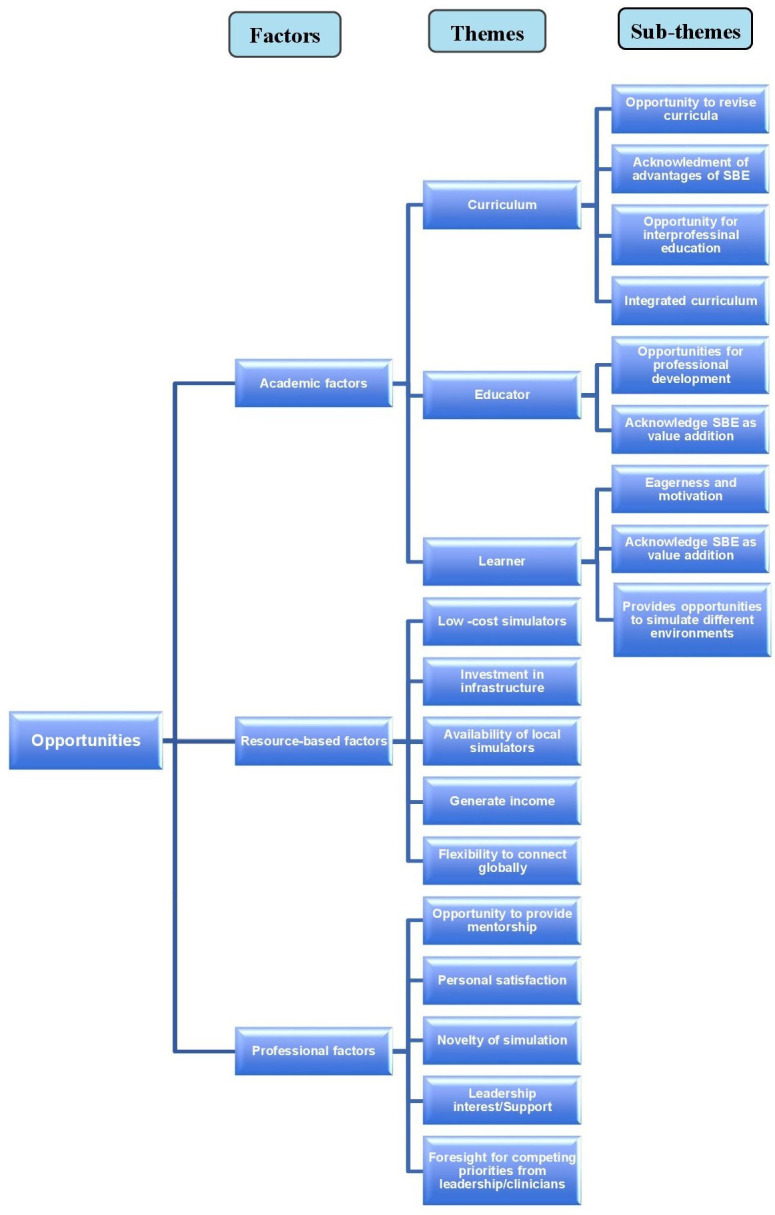
Opportunities identified in the uptake of simulation.

Based on the review of the selected articles, two broad categories of challenges and opportunities were identified. They were then divided into sub-categories of academic, resource-related, and professional factors. Within the sub-categories, there were various themes which are reflected in Figure 2 and Figure 3.

### Challenges

The general category of barriers or challenges faced in the uptake of simulation fell within three sub-categories ( Figure 2). These were:

1. Academic factors (three themes and ten sub-themes)

2. Resource based factors (three themes)

3. Professional factors (three themes)

### Opportunities

The general category of facilitators or opportunities in the uptake of simulation were also divided into three sub-categories ( Figure 3).

1. Academic factors (three themes and nine subthemes)

2. Resource based factors (five themes)

3. Professional factors (five themes)

## Discussion

Simulation aims to avert harm to patients by reducing exposure to inexperienced learners while providing trainees with a ‘substitute’ learning environment that emulates reality for experiential opportunities. Identifying factors impacting use of simulation in health sciences education in the low- and middle-income regions is key to constructing a viable and contextually relevant curriculum that optimizes simulation ^9^. Knowing the benefits of simulation in healthcare education is insufficient on its own until the influencers of success can be categorized, analyzed, and explored comprehensively. Such an approach not only increases the perceived importance but also the potential uptake of simulation in medical education. For the developing world, the barriers and facilitators are often significantly different from those of developed regions; hence, this scoping review is an essential and necessary contribution.

The following sections will describe the opportunities and challenges to the uptake of simulation in low- and middle-income countries in more detail. For ease of understanding, we classified both opportunities and challenges into academic, resources, and professional factors, which highlights the significant interplay between these factors.

### Academic challenges

The category of academic challenges encompasses constraints related to three sub-categories of ‘curriculum design and delivery’, ‘the educator’, and ‘the learner’.

***Curriculum design and delivery.*** At the curricular level, barriers included lack of context-specific curriculum ^10^, difficulty accommodating a simulation-embedded curriculum due to a packed schedule ^11^, limited number of trainers to deliver the curriculum ^4^, and lack of support from leadership to implement a simulation curriculum ^12^. 

Lack of context appropriate curriculum was cited as an important barrier in the implementation of a simulation-enabled/embedded curriculum in low- and middle-income countries in numerous studies ^4, 9– 11^. It is challenging to design and implement a simulation program that aligns with the clinical context. This is a relatively new curricular direction for the developing world which, because of contextual health related issues, requires adaptation or creation of simulation-based case scenarios reflecting conditions of the developing world ^13^. Since there is limited existing literature on simulation in health education in the developing world, it will take some time for these regions to create an effective simulation-enabled/embedded curriculum to address their situational realities ^12, 14^. However, many studies concluded that learning can be better applied when the simulation environment is closer to day-to-day clinical scenarios ^15^.

There are other important curricular barriers to the uptake of simulation, that are more complex and interlinked. Due to lack of finances and limited exposure to technology, curricula have been based on traditional methods of teaching with little flexibility for change and remain content heavy ^16^. Additionally, a limited number of appropriately trained staff, as well as lack of support from the leadership, possibly due to lack of prior exposure and comfort with the conventional pedagogy, have resulted in curricula that are outdated and unable to adapt and respond to contemporary teaching and learning techniques ^17^.

***Educators.*** At the level of the educator, we found that lack of prior use and/or familiarity with simulation contributed to resistance to the uptake of simulation in healthcare education ^9^. These factors contribute to the lack of simulation-savvy human resources. According to a prior study regarding design of simulation-enabled/embedded medical education, lack of qualified simulation instructors was a constraint in the development of a simulation-embedded curriculum ^13^. Educators also feel hesitant in conducting a simulation session if they are not trained prior to it ^15, 18^.

***Learners.*** An academic challenge for learners in adopting simulation was having a high number of fellow students in training programs. This would impact the quality of the exposure the student would get during training ^10^. Additionally, institutional resources in developing regions are limited, often rendering them unable to maintain even the basic needs of trainees, such as libraries and lecture halls. In such contexts, transitioning to a simulation-based curriculum would be challenging, as significant investment in equipment will be required to cater to the large number of students ^4, 16^. This results in the limited ability of healthcare universities in developing countries to adopt new approaches such as simulation ^16, 19^.

Learners may also not be convinced of the pedagogical advantages of a curriculum incorporating simulation ^4^, have experienced simulator breakdown, or have limited accessibility due to equipment deficiencies, which resulted in an unsatisfactory experience ^10, 12^. Moreover, students who participated in simulation activities sometimes described them as stressful and challenging. Interestingly, these feelings were usually reported when they were expected to perform on unfamiliar simulators in front of their peers and teachers ^20^.

***Synthesis.*** We found lack of familiarity with technology and associated performance anxiety are limiting factors for both facilitator and trainee ^16, 17, 21^. Given that most institutes from primary school to university in the low- and middle-income regions have limited financial resources, most students and teachers lack exposure to technology throughout their educational journeys. When such an exposure commences at a later stage, the fear of learning and handling new technologies can be overwhelming, especially when superimposed with curricula which are content-ladened as is common in traditional medical and health science programs ^22, 23^.

### Academic opportunities

***Curriculum design and delivery.*** According to our review, motivation to learn with simulation and acknowledging its ability to add value, competence, and safety served as important facilitators in the development of simulation-embedded curriculum ^17, 18, 24^. The opportunities that a simulation integrated curriculum afforded for increased practice, real time feedback, and clarity in outcomes measured were important factors identified in our review as being facilitators in the uptake of a simulation-based curriculum ^9, 10, 21– 23^.

***Educators.*** If educators recognize the potential of simulation, and offer effective feedback to the institution, the transition to simulation-engaged medical/health provider education will be potentiated ^16, 25^. Faculty desire to develop themselves in simulation-based education, which would increase the availability of experts and mentors in the field, countering the barriers discussed previously ^21^. The momentum towards simulation-engaged/embedded medical/health provider education will eventually lead to an increased number of faculty undergoing training in simulation-based education, providing the opportunity to refine education delivery systems, and to equip educators appropriately for diverse and emergent clinical challenges ^15, 26^.

***Learners.*** There is evidence to show that learners are interested in simulation-based education. This interest stems from the novelty of simulation, the opportunity to practice, and the realization that it promotes patient safety ^27^. This eagerness from the learners will, in turn, encourage faculty to become more acquainted with simulation and to more effectively embed this technology in teaching approaches ^28^.

***Synthesis.*** At the level of both trainer and trainee, an important facilitator was the opportunity to achieve novelty and the acknowledgement that simulation sessions have a positive impact on the ability to diagnose, perform procedures, and integrate basic sciences and clinical medicine ^27, 29^. Trainees appreciate that simulation makes them safer practitioners. It also conveniently provides real time debriefing. The ability to simulate different environments to practice skills is a great advantage ^30, 31^. These are very important factors that motivate facilitators and students and should encourage program and institutional leadership to actively incorporate simulation in curricula ^28, 32, 33^.

### Resources-related challenges

Challenges of resources were broadly encapsulated as financial, infrastructure, and of trained personnel. 

Financial limitation was an important barrier to the use of simulation ^4, 11, 13, 14, 34^. For example, the financial cost to buy simulators was the most important factor identified as a barrier to implement simulation in an undergraduate curriculum in obstetrics ^9^, a finding that was echoed in a study from Ghana as well ^18^. This finding is understandable given the context of low- and middle-income countries which frequently exhibit unstable economies and limited budget allocation to the health sector ^35^. It is routine for hospitals to run out of necessities like medications, syringes, blood pressure apparatus, and oxygen supplies. Likewise, medical colleges often lack up-to-date libraries, access to medical journals, cadavers, anatomy models, and basic technology important for modern student learning ^21, 36^. Under these circumstances, adopting a health curriculum that utilizes simulation may not be a priority for such institutes. Moreover, another associated barrier was maintenance of available infrastructure – the equipment used for simulation is not only costly but incurs ongoing expenses for its maintenance. For many simulators, a specialized staff is required for its handling which adds to overall financial constraints ^37, 38^. The resource challenges do not end at the level of finances. Major electricity blackouts/instabilities and the resultant connectivity issues were reported in our review. In many regions of the developing world, power outages for long periods are not uncommon related to insufficient power production or heavy rainfall which damages the power delivery systems. While hospitals may have backup power generators, these units are focused on lifesaving areas, such as emergency and operating theatres. Such barriers related to infrastructure not only deter the use of simulation but potentially damage simulation related equipment because of abrupt shutdowns or instability ^39, 40^.

Unit cost and operator expertise remain notable areas of concern regarding simulation-based education in resource poor settings ^41, 42^. Inadequate cost reporting is known to occur in simulation related research ^43^. Nevertheless, the availability of modern low-cost simulators could promote self-reliance in LMICs. This is important as it will prevent unnecessary disruptions in the continuity of SBE due to bureaucratic tribulations and will open more avenues for access to simulators ^42^.

Difficulties in coordinating simulation sessions between facilitators and students by administrative staff was a predictable resource challenge in our review ^32, 40, 44^. Limited resources must be allocated to large numbers of students by a small number of staff – which means that multiple simulation sessions must be organized for different groups of students for one topic resulting in problems related to scheduling of students, faculty and space. This finding is reflected in a prior study which also highlighted lack of coordination amongst user groups as an important challenge to simulation in medical education ^40, 42^.

### Resource-related opportunities

While finances are major barriers to simulation-enabled medical education in developing contexts, an efficient economic evaluation can assist in gradually transitioning to a simulation embedded curriculum ^25, 33^. According to a previous study, such an evaluation can inform program leadership and simulation staff on efficiencies in using resources ^23, 25^. Our scoping review found that less expensive equipment can be utilized to meet the target of the curriculum with availability of cheap options in the low to middle income regions when undertaking a hybridization of traditional and sophisticated systems ^45, 46^. Some countries have engaged local industry to manufacture low-cost, contextually appropriate task-trainers ^47, 48^. Tele-simulation is another low-cost intervention that may be successful, as is a computerized patient simulator ^49, 50^. These modifications in content delivery or curriculum will not only improve access to trainees and opportunities to deliver education but can be an income generating opportunity by offering the novelty of simulation related workshops and attracting student intake. Both factors may make it easier and more attractive for an organization to adopt and invest in simulation-based training ^51– 53^. The financial barriers can thus be countered with a long-term approach which transitions into the new curriculum gradually, gathers data continuously, and, as the situation becomes economically feasible, uses the flexibility of the technology to expand. Moreover, utilization of low-cost simulators, electrical generators to provide power, and increasing investments in the sector could potentially mitigate the challenges discussed above ^54, 55^. The increased use of standardized patients for teaching and assessment in resource poor countries is yet another strategy to increase the scope of simulation-based education in these regions ^56^.

### Professional challenges

In terms of professional challenges, our review identified competing professional interests as an important challenge to the uptake of simulation ^57, 58^. Given the context, healthcare facilities in developing regions cater to dense populations with a low healthcare provider to patient ratio ^58^. Furthermore, not all the centers are tertiary care and the ones that are receive an overwhelming number of patients. All these factors essentially mean that most healthcare providers must work beyond their hours to cater to their heavy clinical mandates ^58, 59^. Within this busy schedule, they still try to accommodate teaching via bed side teaching and short lectures ^57, 60^. However, operating sophisticated simulators to conduct teaching sessions requires time and effort to understand the equipment, and faculty may require many practice sessions to become comfortable with the technology ^61, 62^. This results in simulation being under-utilized due to the time required to address the learning curve ^63, 64^. This finding mirrors in a study from Uganda, where the simulation centers faced limited local teaching capacity due to faculty being too busy clinically to devout time to be trained for simulation teaching ^65^.

While simulation in health sciences education decreases the risk for patient and trainees and may have a positive impact on the competence and confidence of the learners, leadership support may be somewhat limited due to a perception that there are limited studies showing its advantages in the ‘real-world’ clinical context ^37, 41, 64^.

Although these challenges might dissuade institutions from establishing a simulation-based curriculum, we also investigated opportunities associated with a simulation-based curriculum that would facilitate the change in education format.

### Professional opportunities

Our review pointed out that the opportunity to provide mentorship and be recognized as a mentor, and a simulationist are important opportunities to increase the usage of simulation ^38, 44, 51^. It is essential that the leadership shows interest and promotes simulation for users to engage more with it ^53, 58^. Trainers indicate willingness to become users of simulation if they were provided protected time and benefits by the academic leadership to enable them to do so ^61– 63^.

Both trainers and trainees reported that peer assisted learning and debriefing, which are standard in simulation-based education are its biggest advantages to increase uptake ^66, 67^. However, faculty needs to be trained in proper debriefing techniques to be truly effective ^68^. The opportunity to teach technical as well as non-technical skills, and the ability to impart remote simulation were factors that trainers found attractive to increase the utilization of simulation-based education in the developing world ^69, 70^.

Professional development of trainers may be possible remotely and in collaboration with simulation societies and provision of scholarships ^58, 65, 70^. Including post-graduate trainees as medical simulation educators may be a good strategy to increase the pool of trainers and increase simulation utilization ^71^. Educators also feel professionally satisfied that they are contributing to a better trained workforce, while improving patient safety, and contributing to better quality of care when imparting simulation-based education. This was a very important motivating factor ^72^.

The scoping review employed rigorous and transparent methodologies throughout the search phase. We systematically explored various electronic bibliographic databases to ensure a comprehensive survey of existing literature. Each citation and article underwent evaluation by two independent pairs of reviewers, who convened regularly to resolve any discrepancies. All citations and articles were accounted for during the process.

Despite our efforts to achieve inclusivity, it's possible that not all scoping reviews in both published and gray literature were identified. Our search strategy encompassed a range of terms; however, alternative terms may also exist. It's worth noting that our search was limited to English terms, which could pose a constraint. Furthermore, we did not engage with researchers or experts to identify additional scoping reviews that may have escaped our attention.

## Conclusions

The developing world faces unique challenges where introducing a new method of education delivery and technology may be questioned. The fact that the conventional curriculum utilizing the traditional method of teaching and training remains in place in LMIC`s suggests that healthcare educators to yet to realize the full potential of simulation-based education. However, the first step is to analyze viability. Through our work, we provided a comprehensive evaluation of the factors that influence uptake of simulation in healthcare education – so that developing contexts can plan the basic framework and gradually prepare to transition into simulation-enabled/embedded environments.

Our review highlighted the key areas of priority that act as deterrents as well as encouragement to the uptake of simulation as a teaching methodology in healthcare education. We feel that it is of utmost importance that academic leadership and faculty show interest and urgency to adopt simulation in curricula and allocate funds for this. Facilitators need to be developed and be provided with protected time to become simulationists. Local manufacturers need to be sourced for simulators, and transfer of technology and expertise needs to be negotiated with vendors. Simulation needs to be looked at from the lens of not only education, but more importantly from one of patient safety in developing countries to allow simulation-based education is mainstreamed in health professions education in low- and middle-income contexts.

### Strengths and limitations

Scoping reviews are utilized to review and understand complex research evidence in areas where there is a dearth of randomized controlled trials. It enables researchers to include a range of different study designs that address questions beyond those related to the effectiveness of a particular intervention. While it provides general information about a research question, further studies are subsequently required to probe specific areas of interest. Within the scope of developing countries, there are variations in socioeconomic conditions which may have affected the conduct and results of the studies.

## Ethics and consent

Ethical approval and consent were not required.

## Data Availability

All data underlying the results are available as part of the article and no additional source data are required. Figshare: Scoping data table. https://doi.org/10.6084/m9.figshare.25706589 ^73^. This project contains the following extended data: Scoping table.doc Figshare: PRISMA-ScR checklist for ‘Challenges and opportunities in the uptake of simulation in healthcare education in the developing world: a scoping review’. https://doi.org/10.6084/m9.figshare.25771791 ^8^. Data are available under the terms of the Creative Commons Attribution 4.0 International license (CC-BY 4.0).
