# Supplementary material for: Challenges and opportunities in the uptake of simulation in healthcare education in the developing world: a scoping review
Source: MedEdPublish (2016). 2024 May 24;14:38. [Version 1] doi: 10.12688/mep.20271.1 (PMC11384200; doi:10.12688/mep.20271.1)
Supplement: Peer review history

# Reviewer report: Reviewer response for version 1

**DOI:** 10.21956/mep.21699.r38593

**Published:** 2024-09-09

**Authors:** Anice George

**Affiliations:** 1 Department of Child Health Nursing, Manipal College of Nursing, Manipal Academy of Higher Education, Manipal, India

This scoping review covers all the aspects as per the objectives of the authors of the review.

 The review has brought out the important aspects on the use of simulation in health care education.

 It would be good to give the country from where the articles were published(For all the 48 articles , in the table containing the results of the reviewed articles).

 In the third paragraph in Introduction "being utilized' is repetition , hence it may be checked and removed.

 A justification on why the benefits of simulation in health care education also may be good.

 Over all this is a well written and presented article, with good information.

Are the rationale for, and objectives of, the Systematic Review clearly stated?

Yes

Is the statistical analysis and its interpretation appropriate?

Not applicable

Are sufficient details of the methods and analysis provided to allow replication by others?

Yes

Are the conclusions drawn adequately supported by the results presented in the review?

Yes

Reviewer Expertise:

Hypertension, Dysmenorrhoea, Yoga, Migraine, Inter-professional education.

I confirm that I have read this submission and believe that I have an appropriate level of expertise to confirm that it is of an acceptable scientific standard.

# Reviewer report: Reviewer response for version 1

**DOI:** 10.21956/mep.21699.r37893

**Published:** 2024-08-05

**Authors:** Cathal Breen

**Affiliations:** 1 Edinburgh Napier University, Edinburgh, Scotland, UK

Thank you for the opportunity to review the manuscript entitled “Challenges and opportunities in the uptake of simulation in healthcare education in the developing world: a scoping review”.

 The review focuses on barriers and facilitators to inform how low to middle-income regions can implement sustainable simulation-enabled medical learning environments.  The PRISMA ScR guidelines were used as the guiding document in the conduct of this study.  The databases, search terms and dates of published work to include are satisfactory.  Overall this work states similar themes found in developed countries that impact the uptake of simulation in healthcare education.  Would it be useful to compare these findings to developed countries as a part of the discussion and conclusion?  I found the article to be satisfactory in content and new knowledge and recommend indexing in your journal. 

 The first sentence of the last paragraph within the “introduction” section reads as so “Scoping reviews are being used increasingly being utilized to review and understand complex research evidence in areas where there is a dearth of randomized controlled trials.  Is there a typo?

Are the rationale for, and objectives of, the Systematic Review clearly stated?

Yes

Is the statistical analysis and its interpretation appropriate?

Not applicable

Are sufficient details of the methods and analysis provided to allow replication by others?

Yes

Are the conclusions drawn adequately supported by the results presented in the review?

Yes

Reviewer Expertise:

I am Professor of Simulation and Clinical skills and my research focuses on heuristics, digital simulation and immersive learning.

I confirm that I have read this submission and believe that I have an appropriate level of expertise to confirm that it is of an acceptable scientific standard.
